# Acromegale

**Published:** 1893-06-24

**Authors:** W. Robertson


### ACROMEGALE.

By W. Robertson, M.D.,
Surgeon Newcastle-on-Tyne Throat and Ear Hospital.

As the name proposed by Pierre Marie, who only so recently as 1886 first clearly isolated the disease, (suggests ἄκρα end, μεγαλη big), Acromegale is characterized by enormous increase in size of the extremities of the body. The hands and feet undergo a remarkable hypertrophy, which in the majority of cases is also well marked in the case of the nose and ears. Another prominent feature is a marked increase in size of the fibro cartilages of the eyelids, nose, ear, larynx, and epiglottis. To the increase in size of the bones of the face, more especially of the superior and inferior maxillæ, is due the elongated elipse the features assume in fully developed cases. The air sinuses of these bones are always more or less enlarged, and contribute towards giving a broader appearance to the face than normal. The hypertrophy in some regions, *e.g.*, the hands and feet, is not alone confined to the skeletal parts, but is demonstrated in all the tissues of the part. The most consistent accompaniment of these external manifestations, whether of a pathological nature, has yet to be determined, is a marked enlargement of the pituitary body, associated with a corresponding depression or excavation in the sella turcica.

From Leontiasis and Osteitis deformans acromegale is clearly demarcated by well-known features clinically, which require no further notice here. The hypertrophy is occasioned by an increase of normal tissue, not an adventitious or inflammatory tissue product.

Such is true for the greater number of cases of the disease where post-mortem investigation has been attainable. It may be interesting to note that in a case of well-established acromegale recently under the care of Dr. Craggs, of the Newcastle-on-Tyne Workhouse, the post-mortem examination revealed a tumour of the pituitary as large as a green walnut.

It cannot be admitted, however, that up to the present this association of enlarged pituitary and acromegale aids much in the elucidation of this strange condition, for as recent literature shows, enlarged pituitary has been observed besides in myxœdema (Boyes and Beadles), cretinism, and cachexia thyreopriva. Enlarged pituitary again has been observed without any of the appearances of acromegale. The division of the pituitary gland, invariably found to be affected is the anterior or glandular portion (in stricture and function similar to that of the thyroid), which is developed from the buccal cavity, and it is interesting in this connection to observe that in acromegale the posterior parts of the mouth, the tonsils, palate, uvula pillars of the fauces, and oropharyngeal mucosa are enlarged, the latter being in some cases thrown up in folds. It ought to be remarked that the several lesions of the pituitary resemble closely those observed in the thyroid. Briefly, the microscope shows "the gland acini of the hypertrophied pituitary dilated, and the colloid matter increased inside as well as outside the acini." The condition of the thyroid is for the most part that of atrophy, as in myxœdema. Apart from the condition of the pituitary and thyroid, the pathological anatomy of acromegale consists in "hypertrophy of all the structures of the part. The sheaths of the peripheral nerves are affected; in the bones a new formation of osseous tissue takes place. The hypertrophy of the tongue is due to an increase of the connective tissue and individual muscle bundles; the sympathetic ganglia in the neck are degenerated; in the atrophied thyroid cystic, and enlarged follicles are observed, while the nasal mucosa is infiltrated.

Another feature in acromegale dwelt upon by certain authors, but less accentuated by many, is an enlargement of the thymus evidenced clinically by a triangular dulness at the upper part of the sternum.

Frequently the appetite for food and drink is large, while the quantity of urine passed is increased. Polyuria and diabetes are frequently observed phenomenon in subjects of the disease. Profuse sweating and headache are frequent symptoms. Age and sex betray little effect on its incidence, while it would seem to affect blacks as well as Europeans. Premature cessation of the menses is often associated with its commencement in women, while in males injuries, accidents, alcoholism, &c., are often predecessors of its development, which is often rapid, and at first refers to swellings of the hands and feet, which are sore and sensitive to the touch.

The skeletal changes present in acromegale are summarised as follows by Thomson (*Jour. Anat. and Phys.*):—

(1) Changes peculiar to acromegale (*a*) enlargement of the pituitary fossa; (*b*) disproportionate development of the bones of the face, dilatation of air sinuses, hypertrophy of cranium clavicles, and enlargement of maxillary articulation. (2) Changes resulting from a tendency to the formation of new bone, both in normal and abnormal sites, thus approaching in character osteo-arthritis. Thus tendons, ligaments, costal, and other cartilages become ossified.

*Inter alia* the external orifice of the external auditory meatus has been found considerably enlarged, rendering the canal funnel-shaped or conical with the apex at the membrana tympani.

From even a cursory review and consideration of all that has been said and written about this most anomalous vagary of nutrition, it is only too evident that but half the picture of the disease is yet visible. It naturally follows, from the fact of the increasing number of necropsies showing enlarged pituitary bodies, that some connection of the nature of cause and effect should be thought of. To this opinion Dr. Marie inclines, and points out that there is a tendency to abnormal development of the osseous system analogous to primary progressive myopathy.

In the alterations in the hypophysis and thyroid, and perhaps also in the persistence and hyperplasia of the thymus, may yet be found the mystery of its causation. The "internal secretions" of these organs (blood glands) exercise an important function in the economy. Abolish the function of the thyroid and myxœdema results. In the case of the pancreas, if this is destroyed pancreatic diabetes supervenes, while if the suprarenals are destroyed a motor paralysis sets in of a nature similar to that effected by curari. The presence of enlarged hypophysis in myxœdema, where the osseous system is never affected, somewhat affects detrimentally the theory that would attribute the causation of acromegale to some chemical substance acting on the tissues as an irritant, its presence being accounted for by the deficiency or entire absence of the internal secretion of one or other of the blood glands mentioned (hypophysis thyroid thymus). Whatever the cause, undue stimulation of nutrition is effected, the excess of this particular function being observed almost in every tissue in the connective, more especially as witnessed in the osseous system.

